# Novel Approaches to Improve the Intrinsic Microbiological Safety of Powdered Infant Milk Formula

**DOI:** 10.3390/nu7021217

**Published:** 2015-02-12

**Authors:** Robert M. Kent, Gerald F. Fitzgerald, Colin Hill, Catherine Stanton, R. Paul Ross

**Affiliations:** 1 Teagasc Food Research Centre, Moorepark, Fermoy, Cork EIRE, Ireland; E-Mails: robert.kent@teagasc.ie (R.M.K.); catherine.stanton@teagasc.ie (C.S.); 2 Department of Microbiology, University College Cork, Cork EIRE, Ireland; E-Mails: g.fitzgerald@ucc.ie (G.F.F.); c.hill@ucc.ie (C.H.); 3 Alimentary Pharmabiotic Centre, Cork EIRE, Ireland

**Keywords:** infant milk formula, pathogens, Cronobacter, manufacturing strategies

## Abstract

Human milk is recognised as the best form of nutrition for infants. However; in instances where breast-feeding is not possible, unsuitable or inadequate, infant milk formulae are used as breast milk substitutes. These formulae are designed to provide infants with optimum nutrition for normal growth and development and are available in either powdered or liquid forms. Powdered infant formula is widely used for convenience and economic reasons. However; current manufacturing processes are not capable of producing a sterile powdered infant formula. Due to their immature immune systems and permeable gastro-intestinal tracts, infants can be more susceptible to infection via foodborne pathogenic bacteria than other age-groups. Consumption of powdered infant formula contaminated by pathogenic microbes can be a cause of serious illness. In this review paper, we discuss the current manufacturing practices present in the infant formula industry, the pathogens of greatest concern, *Cronobacter* and *Salmonella* and methods of improving the intrinsic safety of powdered infant formula via the addition of antimicrobials such as: bioactive peptides; organic acids; probiotics and prebiotics.

## 1. Introduction

Breast milk is recognised as the gold standard [1] in infant nutrition and is consistently recommended by national and international health organizations during the first year of life [2,3]. Breastfeeding provides all the nutrition required for normal infant growth and development, is optimum for infant digestive conditions and also increases the affective relationship between infant and mother. Breast milk contains a number of immuno-regulatory components that lower an infant’s risk of gastrointestinal and respiratory infections. Although the benefits of breastfeeding in providing the optimal balance of nutrients required for infant growth and development are widely accepted and human milk is the first choice for infants, infant milk formulae (IMF) play a vital role when breastfeeding is not sufficient, possible or desirable. Infant formula is intended as an effective substitute and is formulated to mimic the nutritional composition of breast milk. Newborn infants are particularly vulnerable to infection due to immature internal organs and the lack of a developed immune system [4]. Also, commensal microorganism which can act as a barrier to infection in the digestive tract may yet be established at this early stage of life [5]. IMF requires very high levels of microbiological quality and must conform to national and international microbiological criteria [6] due to the susceptibility of newborns to bacterial infection [7]. Unfortunately, current manufacturing technologies mean the generation of a completely sterile product is impossible meaning intrinsic contamination of IMF may be a cause of possibly serious illness in infants [6].

Although production of a completely sterile powdered product is not feasible, every effort is taken to limit the possibility of contamination. High standards of hygiene are maintained throughout the production process and the microbiological quality of each batch is closely monitored; however powdered infant formula (PIF) is not a sterile product even if it has been manufactured to hygienic standards [8] and is not marketed as one [9]. It is, however, a cheaper option than the liquid forms of feed and has a longer shelf life. From a manufacturers’ perspective, it offers a greater scope for modifying the formula density should it be required during production [10]. Processing methods and concerns for the microbiological quality used for standard PIF also apply to formula designed for ill children and also follow-up formula (FUF).

In this article, we review PIF and the problems associated with bacterial contaminations, particularly contamination by *Cronobacter* and *Salmonella*. There is an increasing interest in the development of natural effective antimicrobial agents for the preservation of foods due to safety concerns with synthetic compounds [11]. Specifically, there is an increasing interest in the use of natural antimicrobials for use in PIF with direct action against *Cronobacter* [12]. For this reason, we will also discuss the potential of natural additives capable of preventing the proliferation of disease causing pathogens in formula, and how such strategies can be implemented with regard to the strict infant formula legislation in place.

## 2. Production of Powdered Infant Formula

Bovine milk is the basis for most infant formulae but contains higher levels of fat, minerals and protein compared to human milk so it is skimmed and diluted, to more closely resemble human milk composition [13,14]. Other animal milk based formula are currently not acceptable, principally due to a lack of robust randomized clinical trials (RCTs) [15]. Soy based formulae are also available and are widely used, but it has been recommended that their use be limited due to potentially harmful effects to the infant due to the presence of phytoestrogens in the formulae [16,17].

The composition of PIF is strictly regulated and each manufacturer must follow established guidelines outlined to them by governing bodies. These values are set by the Codex Alimentarius Commission (CAC 1979), company-company agreements as well as “in-house” levels regarded as workable based upon prior experience [7]. All the major components added to formula (protein, lipids, carbohydrates) have minimum and maximum values (Table 1). Ionizing radiation is not permitted due to the organoleptic deterioration it would cause the product [13,18]. Both whey and casein are acceptable sources of protein, and individual amino acids may be added to improve the formula’s nutritional value. However, only L forms of these amino acids are allowed while D forms are not permitted for fear they may cause d-lactic acidosis [19]. Commercially, hydrogenated fats and oils are not allowed. Fructose is avoided due to hereditary fructose intolerance present and undiagnosed in some newborns, which may have fatal consequences. Many vitamins and minerals have a guidance upper level (GUL) amount for inclusion in PIF. These levels are designed for nutrients without enough information for a full rigorous risk assessment. These components must have an established history of safe use, and aid in the infant’s nutrition [13]. The required range of each nutrient must be maintained throughout the shelf-life of the product [20]. Of note is that infant formula is currently the only food product mandated by the United States Food and Drug Administration (FDA) to print a use by date on the product packaging. Prolonged storage can lead to settling of contents and subsequent blockage of the milk bottle nipple [21].

In early infancy, allergy to milk is the most common food allergy reported, affecting 2%–3% of children. Symptoms associated with food allergy may include immunoglobulin E (IgE) associated reactions (such as wheezing and eczema) and non-IgE associated reactions (such as esophagitis) [22] Proteins present in milk are recognised as the main cause of allergic reaction [23,24]. Formula intended to be hypoallergenic must be tested in infants with hypersensitivity to cow’s milk and properly verified by elimination challenge tests [22]. Hypoallergenic infant formula can be generated via enzymatic hydrolysis and heat-denaturation of the intact proteins in order to generate smaller molecules with reduced atopic potential [25,26,27]. The resulting formula may contain hundreds of smaller peptide fragments following this treatment [23]. Hypoallergenic formula must fulfill the infant’s nutritional requirements in the same manner as standard infant formula. In order to achieve hypoallergenic status, the product must demonstrate zero allergic symptoms in 90% of infants with confirmed cow’s milk allergy when given in well controlled, scientifically robust clinical trials [22]. In the case of infants who demonstrate hypersensitivity to hydrolyzed protein based formula, amino acid-based formula are now available. These products are typically very expensive [20,28].

PIF can be produced in one of three different ways, a dry-mix, wet-mix or combined process. In the dry-mix process, the various formula components are received heat-treated separately from suppliers and blended together to ensure a uniform blend of nutrients throughout the powder. This process is less capital intensive than the wet-mix process and importantly, allows the manufacturing line to be maintained in a dry state for prolonged periods of time, denying any pathogens present access to water and thereby minimizing the chance of bacterial growth. The microbiological quality of the finished product is determined by the individual component ingredients received by the manufacturer as in many cases the powder will not receive further in-house heat-treatments. For this reason, PIF manufacturers who use the dry-mix method try to develop and maintain close relationships with their suppliers. After large-scale blending of the individual components the final product undergoes a final check to ensure conformance to appropriate specifications.

**Table 1:** The minimum and maximum values (/100 kcal) of the major components added to infant milk formula (adapted from [13]).

Nutrient	Minimum	Maximum
Protein (g)	1.8	3
Taurine (mg)		12
Lipids (g)	4.4	6
Linoleic acid (mg)	300	1200
Phospholipids (g)		2
Carbohydrates (g)	9	14
Fructooligosaccharides/Galactooligosaccharides *		0.8 g/100 mL
Sodium (mg)	20	60
Potassium (mg)	60	160
Chloride (mg)	50	160
Calcium (mg)	50	140
Phosphorous (mg)	25	90
Magnesium (mg)	5	15
Iron (mg)	0.3	1.3
Zinc (mg)	0.5	1.5
Copper (μg)	35	100
Iodine (μg)	10	50
Selenium (μg)	1	9
Manganese (μg)	1	100
Fluoride (μg)		100
Vitamin A (μg-Retinol equivalent)	60	180
Vitamin D (μg)	1	2.5
Vitamin K (μg)	4	25
Vitamin E (mg α-Tocopherol equivalent)	**	5
Vitamin C (ascorbic acid) (mg)	10	30
Vitamin B1 (thiamine) (μg)	60	300
Vitamin B2 (riboflavin) (μg)	80	400
Vitamin B6 (pyroxidine) (μg)	35	175
Vitamin B12 (μg)	0.1	0.5
Niacin (μg)	300	1500
Folic Acid (μg)	10	50
Pantothenic acid (μg)	400	2000
Biotin (μg)	1.5	7.5
Choline (mg)	7	50
Inositol (mg)	1	10
cytidine 5’-monophosphate (mg)		2.5
uridine 5’-monophosphate (mg)		1.75
adenosine 5’-monophosphate (mg)		1.5
guanosine 5’-monophosphate (mg)		0.5
inosine 5’-monophosphate (mg)		1

* shall not exceed 90% oligogalactoysyl-lactose and 10% high molecular weight oligofructosyl-saccharose; ** 0.5 g of polyunsaturated fatty acids expressed as linoleic acid as corrected for the double bonds but in no case less than 0.5 mg per 100 available kcal.

A wet-mix process involves blending, homogenising and pasteurizing all the components together. Due to the inclusion of a heat-treatment step the microbiological quality of the finished product is far less reliant on the base ingredients. This process also ensures the uniform distribution of nutrients throughout each batch. The nature of this process requires that manufacturing equipment be routinely wet-cleaned. The presence of liquid in the manufacturing environment due to this cleaning can allow the growth and establishment of microorganisms. Manufacturers separate their plants into strict wet and dry areas and restrict movement of equipment and personnel in these areas in an effort to minimize cross-contamination. Following pasteurization heat-sensitive micronutrients are added. The microbiological quality of these micronutrients is important as further heat-treatments may not be applied to the product. The liquid mix is then spray-dried to form a powder.

Lastly, the Combined process involves drying a wet mixture of the major formula ingredients (fat, protein and carbohydrates), referred to as infant formula base powder. This base powder is then combined with pre-dried minor ingredients (vitamins and minerals) and mixed to produce the finished product. Regardless of the process used the resultant powder is packed in containers, flushed with inert gas, sealed with an airtight cap, coded and labelled. Each batch of formula is usually held until samples from it have undergone analysis for uniformity, nutritional content and microbiological safety [18].

Various food safety authorities regulate and monitor the components and manufacture of PIF and scientific committees advise on whether ingredients should be added and/or removed or whether the amounts of individual components should be altered with regard to new research. For instance, the Codex Alimentarius Commission (CAC) develops and adopts food standards that serve as reference for the international food trade. It comprises 186 members (185 states and 1 organisation (European Union, EU)). In addition, over 200 non-governmental organisations and inter-governmental organisations are observers of the CAC. In many cases Codex standards also serve as a basis for national legislation [29]. For EU member states the EU directive 2006/141/EC (this directive was recently slightly amended with regard to protein content (Commission directive 2013/46/EU)) sets values and labeling protocols for the composition of infant formulae and follow-on formulae.

It is important to emphasize that even if perfectly sterile processes conditions were achieved in a factory setting, exposure is more likely to come from the formula preparer/caregiver [18]. For example, extrinsic contamination can occur due to use of contaminated utensils and equipment in the preparation environment or from the preparer and can influence the microbiological safety of both powder and liquid infant formulae. In 2007 the World Health Organization (WHO) in collaboration with the Food Safety Authority of Ireland (FSAI) and the Food and Agricultural Organization of the United Nations (FAO) issued guidelines for the safe preparation, handling and storage of PIF. If the formula preparer follows such guidelines correctly the potential for infection will be low [8]. However in certain instances, through carelessness or incompetence, appropriate guidelines may not be followed. The presence of a microbiological protectant in the formula could help maintain a low amount of a pathogenic organism following reconstitution and lower the risk of catastrophic infection. While microorganisms do not grow in dry PIF, following reconstitution it provides an excellent medium for bacterial growth particularly if it is not refrigerated. If held for an extended period of time certain microorganisms present in the formula, either due to extrinsic or intrinsic contamination will rapidly grow to potentially harmful levels.

## 3. Pathogens in Infant Formula

### 3.1. Introduction

As previously mentioned PIF is not a sterile product. Milk feeds act as excellent media for bacterial multiplication so any pathogens which have survived processing or contaminate the product afterward may rapidly multiply if given the opportunity post rehydration [10]. A joint FAO/WHO consultation group (2004–2006) identified the primary microorganisms associated with PIF contamination as *Cronobacter* sp., *Salmonella enteritidis*, *Enterobacter agglomerans*, *Hafnia alvei*, *Klebsiella pneumoniae*, *Citrobacter koseri*, *Citrobacter freundii*, *Klebsiella oxytoca*, *Enterobacter cloacae*, *Escherichia coli*, *Serratia* sp., *Acinetobacter* sp., *Bacillus cereus*, *Clostridium difficile*, *Clostridium perfringens*, *Clostridium botulinum*, *Listeria monocytogenes* and *Staphylococcus* sp*.* *Cronobacter* sp. and *Salmonella enterica* were identified as the pathogens of most concern in PIF. PIF contaminated with either of these organisms has been associated with serious illness and death [8].

### 3.2. Cronobacter

*Cronobacter* are gram-negative, motile, non-spore-forming peritrichous rods of the *Enterobacteriaceae* family [30,31]. Previously referred to as *Enterobacter sakazakii*, the microorganism has recently been formally reclassified as a new genus, *Cronobacter*, currently comprising ten species, *Cronobacter* *sakazakii*, *Cronobacter malonaticus*, *Cronobacter turicensis*, *Cronobacter muytjensii*, *Cronobacter dublinensis***,** *Cronobacter universalis*, *Cronobacter condimenti*, *Cronobacter helveticus*, *Cronobacter pulveris* and *Cronobacter zurichensis* [32,33,34,35]. Due to the relatively recent reclassification, there is uncertainty regarding the specificity of *Cronobacter* in publications prior to 2007. The species can be classified into two groups: Group 1 (*C. sakazakii* and *C. malonaticus*) and Group 2 (*C. turicensis* and *C. universalis*). From a healthcare perspective Group 1 species are of more importance and form the majority of clinical isolates [35].

*Cronobacter* infection represents a serious health risk in infants; particularly neonates, and death may occur in 40%–80% of cases [36,37,38]. The microorganism has been implicated in cases of meningitis, septicemia, and enteritis and is found in sites normally sterile in healthy individuals such as blood, bone marrow and cerebrospinal fluid in infected individuals [33,36,37]. *Cronobacter* infection has also been implicated in cases of necrotizing enterocolitis (NEC) which affects 2%–5% of all premature infants and is the most common gastrointestinal emergency in neonates [39,40]. Infected infants are usually treated with antibiotics such as ampicillin/gentamicin or ampicillin/chloramphenicol [41]. Survivors often suffer from various neurological and/or gastrointestinal sequlae such as hydrocephalus, poor neural development [42] and short gut syndrome [43]. Since 2002 *Cronobacter* have been recognized as “Severe for restricted populations, life threatening or substantial chronic sequlae for long duration” by the International Commission on Microbiological Specification for foods (ICMFS) [7,44] An infectious dose of 10,000 Colony Forming Units (CFU) in a single feeding was proposed by the WHO/FAO in 2007 (WHO/FAO 2008). Although understanding of *Cronobacter* has increased considerable in recent years, there remain unresolved questions regarding its pathogenicity and few virulence factors have been identified to date.

It is likely that the infection route is via attachment and invasion of cells in the intestine following consumption. Indeed, gene clusters responsible for fimbriae have been identified in a number of *Cronobacter* genomes. *C. sakazakii* species possess genes which encode for Beta-fimbriae while other *Cronobacter* genomes encode for curli fimbriae [45,46] which may reflect a host-influenced evolution [35]. *C. sakazakii* alone also utilizes exogenous sialic acid, a substance present in breast-milk, mucin and gangliosides (glycosphingolipids associated with the central nervous system) which is added to infant formula due to its association with brain development [47]. Five putative type VI secretion system (T6SS) gene clusters have been recognized in *Cronobacter* genomes although functional expression of these genes in *Cronobacter* is yet to be confirmed [45,48,49]. This system is recently described and may be involved in cytotoxicity, cell adherence/invasion and the ability to survive within the host. A number of studies have indicated that outer membrane proteins (omp) A and X have roles in invasion and translocation of brain microvascular endothelial cells which form the blood-brain barrier [50,51]. The mechanism(s) responsible for brain cell destruction are yet to be elucidated [35].

The natural reservoir of *Cronobacter* has not been confirmed. It has been isolated from both human and non-human sources, with PIF the food most commonly associated with infection. However the source of contamination of PIF by *Cronobacter* is an area of contention [52,53]. The bacterium has been isolated from home [54] and hospital environments [55] as well as milk powder processing plants [56,57]. *Cronobacter* has also been detected in previously unopened PIF products indicative of intrinsic contamination [8,18,58]. Certain components of PIF such as starches and inulin have been considered as possible sources of contamination [59]. Although it is generally agreed that *Cronobacter* are relatively thermotolerant organisms, they are incapable of surviving pasteurization [60] indicating that PIF becomes contaminated downstream of this process [37,38,42] by additional ingredients, plant equipment or via asymptomatic carriage of *Cronobacte*r by workers in the processing plant. Indeed *Cronobacter* has been isolated from the faeces, skin and mouth of otherwise healthy individuals [61,62].

Regardless of how contamination occurs, once present it is estimated that some strains of *Cronobacter* can survive, dormant in PIF for at least two years [42] and rapidly grow upon reconstitution [63]. Tolerance of desiccated conditions for such an extended period of time can be attributed to certain aspects of the bacterium’s physiology, perhaps most notably the propensity some strains possess to produce a capsule [35,63]. *Cronobacter* is resistant to desiccation over a wide range of a_w_ (0.25–0.86). Over a 12 month storage period, the pathogen has been shown to survive better in dried formula with an a_w_ between 0.25 and 0.30 than at 0.69 and 0.82 [31]. This resistance to osmotic stress may be aided by the organisms ability to accumulates solutes such as trehalose which can help stabilize membrane components [64]. As well as protection from desiccation, the capsule material may also protect *Cronobacter* from disinfectant agents [65] used to clean processing plant equipment and preparatory utensils. Upon reconstitution, the organism is capable of growth at temperatures from as low as 6 °C to as high as 47 °C [66]. *Cronobacter* can adhere to materials commonly used in food preparation utensils (e.g., silicone, stainless steel and polycarbonate) which may increase the chance of extrinsic contamination during formula preparation and feeding [35]. Finally, the organism appears to tolerate a broad range of pH conditions (pH 4.5–10) which contributes to its survival under various acidic/basic conditions [64].

The rise in *Cronobacter* notoriety has prompted changes in the microbiological criteria for PIF and reconstitution procedures. Perhaps most notable is the FAO/WHO document [8] “Guidelines for the safe preparation, storage and handling of powdered infant formula” which was the result of two FAO/WHO meetings held in 2004 and 2006 [53]. The document recommends reconstituting with water which has cooled to 70 °C from boiling in order to destroy vegetative cells, reconstitute only the required amount and reduce the storage period prior to consumption as much as possible [8]. The use of 70 °C water is not adhered to in all countries and can be impractical for a number of reasons. For one, the insertion of a thermometer into the liquid presents another route of possible for contamination. For this reason it is advised to boil water and let it cool for 30 min which can be impractical (particularly in cases where feeding may be required every two hours) and inexact due to extrinsic influences on temperature. The use of 70 °C (or higher depending on the accuracy the caregiver practices) water may also affect the nutritional quality of the formula due to damage to heat sensitive ingredients such as some vitamins and probiotics [35].

Various microbiological tests are applied to samples from each PIF batch which are compared with microbiological criteria outlined by the CAC, “in-house” levels deemed workable due to prior experience and company agreements [53]. Many infant food producers are legally-bound to implement good manufacturing processes (GMPs) or Hazard Analysis Critical Control Point (HACCP) principles into their control plan in order to reduce the risk of contamination [67]. In the USA, only the state of Minnesota requires reporting of incidences of *Cronobacter* infection [9,68]. The incidence rate of infection for the USA are at least one per 100,000 infants which rises to 9.4 per 100,000 in infants of low birth weight [58]. A 2002 FDA study which detected *Cronobacter* in 23% of sampled PIF [9], coupled with three neonatal intensive [40,52,69] care outbreaks which strongly implicated PIF as the vehicle of transmission prompted the WHO to announce that PIF had strong links microbiologically and epidemiologically to *Cronobacter* infection in infants [18]. The previous FDA method for analysis of PIF was based on the most probable number (MPN) approach which was then followed by a number of culturing steps which could take a week to produce a result. Presumptive *Cronobacter* isolates were then confirmed using biochemical profiling (API) and oxidase tests. In March 2012 the FDA replaced the chapter “Isolation and Enumeration of *Enterobacter sakazakii* from Dehydrated Powdered Infant Formula” with a chapter specifically for *Cronobacter* in their Bacteriological analytic manual which can be located at (http://www.fda.gov/Food/FoodScienceResearch/LaboratoryMethods/ucm289378.htm) [70]. The new method includes Real-Time Polymerase Chain Reaction (RT-PCR) analysis for rapid screening of isolates as well as more traditional culture-dependent ones. Positive RT-PCR should be validated using the cultural method [60]. The FDA are currently implementing new rules for PIF manufacturers which include specific testing for *Salmonella* and *Cronobacter* [71].

Outbreaks of neonatal *Cronobacter* infections are hard to document due to the difficulty in proving that it was contaminated PIF that caused the illnesses. There currently exists no global active surveillance network for this pathogen. The FAO/WHO reported 120 incidences of *Cronobacter* in children younger than 3 years old between 1961 and 2008 [72]. It is estimated that the actual level of infection is higher [73]. Specific incidences of infant and neonatal *Cronobacter* infection have been recently reviewed elsewhere [53]. It should be noted that the outbreak [52] which initiated the FAO/WHO risk assessment of *Cronobacter* and the WHO preparation of PIF guidelines was due to a formula product specifically for neonates and not regular PIF and as such, is not covered by any new legislation related to standard PIF [35]. Also of note is the fact that in certain instances, children below 6 months of age receive follow-up formula (FUF) in error [5]. Changes to PIF legislation should accommodate this malpractice.

### 3.3. Salmonella

*Salmonella* diverged from a common ancestor with *Escherichia* 100–150 million years ago and evolved as an intracellular pathogen [74]. The taxonomy and nomenclature of *Salmonella* are controversial, complex and continually evolving. The *Salmonella* genus is composed of two distinct species, *Salmonella bognori* (a cold-blooded vertebrate commensal) and *Salmonella enterica*, which is divided into six sub-species. These sub-species are classified in to 50 serogroups based on the somatic (O) antigen, and further divided into over 2400 serovars based on the flagellar (H) antigen. The majority of serovars associated with diseases in humans belong to *S. enterica* subsp. *1*. This subspecies causes two types of diseases in humans due to ingestion of contaminated foodstuffs or water, enteric fever (typhoid) or gastroenteritis. Gastroenteritis is mainly caused by *S. enteritidis* and *S. enterica* serovar *Typhimurium* [75]. Intrinsic contamination of PIF with non-typhoidal *Salmonella enterica* is an important cause of infection and illness in infants [18].

Rates of *Salmonella* infection are highest in infants less than a year old [76]. In the United States for example, incidence rates in infants are eight times higher that rates observed in the adult population. The higher incident rate may be due to a higher susceptibility in infants, or the fact that infants are more likely to receive medical attention than adults [58]. Infections for the most part are sporadic [77] but outbreaks caused by the pathogen have been recorded [78,79]. Outbreak detection is usually due an unusual serotype of *Salmonella*, possessing a trait facilitating their detection being responsible for illness (FAO/WHO, 2006). Non-typhoidal *Salmonella* infection can cause gastroenteritis leading to diarrhoea, fever and vomiting [76] but in certain instances can lead to more serious conditions such bacteraemia, septic arthritis, pneumonia and meningitis. Due to their immature immune systems, infants infected by Non-typhoidal *Salmonella* are more likely to suffer these debilitating potentially fatal diseases [76,79]. The severity of disease *Cronobacter* can cause in infants, coupled with its relatively recent emergence has caused it to overshadow concerns regarding contamination of infant formula with *Salmonella* [78]. It should also be noted that in due to the nature of bacterial transmission which can occur when an infected individual practices poor hygiene, non-infant formula caused illness is far more likely than illness caused by intrinsically contaminated PIF. When intrinsic *Salmonella* contamination does occur, the bacteria generally enter infant formula due to contamination by ingredients which have not undergone sufficient heat-treatment, or contamination due to the processing environment post-thermal treatment [18].

Control measures implemented by PIF manufacturers are similar to those used by other dairy powder manufacturers and are based on four principles: (1) Avoidance of entry of *Salmonella* into the processing facilities and in particular the zones from drying to filling, considered as high-hygiene area (appropriate zoning and segregation); (2), avoidance of multiplication of *Salmonella* in case of entry (elimination of water); (3) hygienic design of high-hygiene zones and the equipment located in such zones; and (4) use of dry-mix ingredients which are free of *Salmonella*.

*Salmonella* infections and outbreaks due to intrinsically contaminated PIF are usually only reported in instances where the causal strain is a rare one. At least five outbreaks related to intrinsic PIF contamination occurred between 1985 and 2005. An outbreak occurring in France in 2005 led to 141 confirmed cases of illness. A case controlled study conducted to find the source of the infection identified a particular infant formula brand as responsible and molecular identification techniques provided verification [80]. This case highlights the importance of surveillance, which in France is based on a network of laboratories which voluntarily send isolates to a national reference centre to be serotyped. The rare serotype *S. enterica* Agona, was implicated and allowed the source of infection to be traced. In 2008, Spain’s National Centre of Microbiology notified an increase in *S. kedougou* isolates from ill children leading to a product recall for the infant formula implicated. During the outbreak 31 children (>1 year) became ill and symptoms included fever and diarrhoea. *Salmonella* isolated from the patients showed indistinguishable pulse-field gel electrophoresis (PFGE) profiles and identical antibiotic resistance profiles. The study indicated that a particular brand of PIF was the cause of the outbreak and no further cases were detected following its recall. The strain implicated had previously been responsible for two outbreaks in the United Kingdom and Norway (meat products were implicated in those cases). The authors concluded that the increase in this serotype of *Salmonella* was only detected due to the low-expected frequency of it in Spain [81].

Current Codex Alimentarius recommendations indicate a product is fit for consumption if sixty 25 g samples test negative for microorganisms. However, *Salmonella* is rarely detected in PIF using this method [81]. In one outbreak in 1985, it was reported that *Salmonella* was present in very low numbers (1.6 organisms per 450 g) [58]. In most investigations, the epidemic strain is isolated from bulk samples. Outbreaks of salmonellosis due to contaminated PIF are likely underreported due to the difficulty in implicating a serotype without a rare trait, a lack of routine serotyping in an area or blaming an illness on another factor [58].

## 4. Antimicrobial Agents

As current technologies prohibit the generation of a completely sterile PIF product, researchers are currently investigating a number of methods to improve microbiological safety via inclusion of intrinsic protectants. The most promising approaches have are described in the following section.

### 4.1. Bioactive Peptides

Bioactive peptides are small peptide sequences consisting of 3–50 amino acid residues [82,83]. These peptides may possess a number of physiological properties including opiate, anti-thrombotic, anti-hypertensive and antimicrobial activity *in vivo* and *in vitro* [84]. These peptide sequences exist in a latent, encrypted state in a precursor protein which may be of animal, bacterial or plant origin [85] and are released via direct enzymatic hydrolysis or fermentation [82]. To date, hundreds of bioactive peptides have been identified and this number continues to grow as novel methods of generating peptides are developed [83,86]. Milk proteins have been recognised as one of the most valuable sources of bioactives [82,87] and are generally assured to be safe and inexpensive [83]. Particular to this article, from a regulatory standpoint, the inclusion of dairy-derived bioactive peptides in PIF is not of major concern. As previously described in Section 2 of this article, hydrolysed milk protein fragments are already present in formulae in the form of hypoallergenic infant formula [88].

Milk is composed of a number of proteins, the major two being bovine casein and whey with casein accounting for 80% of the total protein content [89]. There are 4 major types of casein proteins, αS1-, αS2-, β- and κ-casein [90]. Whey protein consists of a number of globular proteins such as bovine serum albumin and β-lactoglobulin, isolated from the whey fraction of milk [91]. Some whey proteins, such as lactoferrin possess inherent antimicrobial properties [83,92].

Antimicrobial peptides (AMPs) have been recognised as important elements of the innate immune system [93]. These peptides are diverse and ancient molecules and are present in various sources. Yet, regardless of the source, antimicrobial peptides show similar recurrent structural and functional characteristics. These peptides interact with bacterial membranes causing disruption leading to rapid cell death [94]. Most AMPs can adopt an amphipathic conformation allowing for interaction with hydrophilic and hydrophobic interfaces on the bacterial membrane. Peptides also contain a positively charged domain which aides disruption of the negatively charged bacterial membrane [95]. It is thought that AMPs could also target intracellular components of bacteria such as nucleic acids or intracellular proteins [96]. To date, physiologically active peptides such as those possessing angiotensin-1-converting enzyme (ACE)-inhibitory activity have been the subject of more scrutiny than antimicrobial peptides [83]. In the past, healthcare industries had an initial lack of interest in antimicrobial peptides due to cost and efficacy in comparison with conventional antibiotics [97]. However, due to a consumer preference for lightly processed foods containing low-levels of artificial preservatives and chemicals, AMPs are now being investigated as possible natural bio-preservatives [98]. Although current antimicrobials may not be potent enough to use directly as a replacement for traditional antibiotics, the potential to act as a natural prophylactic protectant in many foods cannot be underestimated [83].

One of the best known AMPs is Lactoferrin (LF), which is an 80 kDa iron-binding protein [94,99] found in neutrophils and most other biological exocrine secretions. It is present in the whey component of milk of a number of mammals [100]. LF is resistant to proteolysis in the gut and can be found intact in the faeces of breastfed neonates signifying that initially, it may not have a large role to play in infant nutrition [101,102]. The concentration of LF in human milk is 2 mg/mL (although this varies during lactation, at points up to 10 mg/mL in colostrum [103]) compared 0.02–0.2 mg/mL in bovine milk [104] indicating it’s importance to infants. The iron-binding activity of LF allows it to inhibit the growth of bacteria, parasites and fungi [105]. LF is also capable of binding to the surface of Gram-negative bacteria causing cell death due to the release of lipopolysaccharide (LPS) from the cell membrane [106] which occurs in a temperature dependent manner [98]. Apart from antimicrobial properties, the bioactivities of LF include anti-inflammatory, anti-cancer and immuno-modulating properties as well as the ability to promote the growth of *Bifidobacteria* [107].

A recent study evaluated the antimicrobial activity of LF (of bovine origin) on *Cronobacter*. The authors used both native and iron-saturated LF in their antimicrobial assays which were carried out in phosphate buffer, bovine whey and bovine skim milk. The results suggest that binding of iron is important for antibacterial activity against the pathogen as native LF was the only form which reduced growth (concentration and incubation time-dependent) in all media tested. The study also demonstrated the protective effect that skim milk and whey have for the bacteria in comparison to the phosphate buffer which is of importance with regards to PIF. The authors also concluded that bovine LF is highly resistant to most standard forms of pasteurization [12]. Another study demonstrated the anti-adhesive effect that LF had against a *Cronobacter* strain originally isolated from PIF (*C. sakazakii* 4603) as intestinal adherence is an important initiator of bacterial pathogenesis [108,109]. The results indicated that adherence could be significantly reduced (80%–99%) *in-vitro* at a concentration of 10 mg/mL LF. The inclusion of an oligosaccharide did not increase anti-adhesive activity [110]. Of note, a study by Al-Nabulsi *et al.* demonstrated that PIF may have an inhibitory effect on LF antimicrobial activity, possibly due to the high concentration of divalent cations in the powder [98] which has been shown to reduce the antimicrobial activity of LF against other pathogens [106]. The authors found that 2.5 mg/mL of LF was able to inactivate 4 log 10 CFU/mL of undesicated *Cronobacter* cells when suspended in peptone water at 37 °C. However, there was no detectable antimicrobial activity in PIF under the conditions tested. The authors also concluded that the high concentration of iron in reconstituted PIF may have negated the iron binding ability of LF [98]. In addition to the antimicrobial effects exhibited by LF, studies have shown it can have a positive effect on outcomes of respiratory disease and levels of red blood cells in circulation when added to infant formula [15,111]. Recently, an EFSA panel announced that bovine LF is safe for inclusion in infant formula at specific concentrations [112].

In 1992, Bellamy *et al.* [113] identified an *N*-terminal region within LF that was antimicrobial. Pepsin digestion of both human and bovine LF, leads to the release of the molecule lactoferricin human (LFcinH) and lactoferricin bovine (LFcinB) respectively. Like LF, LFcin possesses antimicrobial activity against a variety of Gram-positive and Gram-negative microbes. Indeed, it is suggested that the molecule’s smaller size allows better access to bacterial membranes increasing potency [114]. In this respect, it can directly cause bacterial death complementing the iron chelating-mediated antimicrobial activity associated with intact LF [115]. LF has shown antimicrobial activity against a broad range of pathogenic bacteria, either via iron sequestering or direct bacterial membrane interference [116]. Methods for the production and pasteurization of both LF and LFcin have been patented and both have been added to a number of foods including infant formula to improve safety [92]. A third protein, within the LF parent sequence, lactoferrampin has also shown broad spectrum activity against both Gram-positive and Gram-negative bacteria and the yeast *Candida albicans*. Lactoferrampin is found within the N1 domain of bovine lactoferrin and appears to be crucial for the candidacidal activity of the parent protein [117].

The αS1-casein component of casein has been shown to be a source of a number of AMPs. A notable and early example is isracidin, produced by the chymosin-mediated hydrolysis of bovine casein. The peptide possesses bactericidal activity against a broad range of pathogenic Gram-positive and Gram-negative bacteria. The Weizmann Group, the discoverers of isracidin had previously isolated and patented some of the earliest AMPs, the casecidins. Like isracidin, the casecidins had a broad spectrum of activity, particularly against Gram-positive bacteria [97]. The casecidins required high concentrations *in vitro* in order to be effective and so were not seen as effective antimicrobials [83,97]. Similarly, isracidin also requires high concentrations *in vitro* [118]. However, *In vivo* experiments with the peptide have shown it to be more effective however. Isracidin was shown to be non-toxic and effective in preventing illness due to *S. aureus* infection in a range of animals and an effective prophylactive anti-mastitis treatment over a long period similar to an innate immune-response [97]. The isracidin case highlights differences in potency of antimicrobial peptides *in vivo* and *in vitro* and the importance of possible indirect modes of action which help prevent illness in the body [83,97,119].

In addition, Hayes *et al.* [118] described the generation of 3 AMPs from αS1-casein. These peptides, namely caseicin A, B and C were generated through fermentation. Caseicin A and B share homology with isracidin consisting of 9 and 8 amino acid residues respectively, found within it. Results from *in vitro* assays indicated that these peptides (particularly caseicin A) had potencies similar to that of isracidin against *E. coli* and were also effective against other Gram-negative bacteria but less so against Gram-positives. Caseicin A possesses a +2 positive charge and has a lower minimum inhibitory concentration (MIC) value than the neutral caseicin B. Caseicin C is the least potent of the three described [118]. A second study by Hayes *et al.* [120] investigated the possibility of producing a safe antimicrobial agent capable of inhibiting the growth of *Cronobacter* in infant formula. The study used a filtered (3 kDa) fermentate derived from sodium caseinate containing both caseicin A and B peptides and added it to PIF at various concentrations. At 0.2% (wt/vol) the antimicrobial agent had a bacteriostatic effect on the pathogen and at higher concentrations, a bactericidal one.

Casocidin-1 is a 39 amino acid sized fragment originating from bovine αS2 casein, characterised in 1995, which possesses antimicrobial activity against a range of bacteria including *Bacillus*, *E. coli* and *Staphylococcal* strains [121]. It was proposed that this peptide could be used in a number of food products including infant formula as a preservative with antibacterial properties. It has not been used commercially due to the difficulty in producing a pure product. It has been proposed that a crude milk preparation containing casocidin-1 would be a better alternative to a pure product in order to ease production [83]. In fact it was reported that various fractions of a crude preparation possessed some antimicrobial activity suggesting a synergistic effect between a number of factors [121].

K-casein has also been a source for antimicrobial bioactives, most notably kappacin, also known as caseinomacropeptide (CMP). CMP exists in a number of variations but is active only in a nonglycosyltated, phosphorylyated form [90]. Kappacin was patented [122] and has been used commercially as an oral hygiene product in its pure form and also in a combination with zinc (which enhances its antibacterial activity). It has been proposed that kappacin could also be used as a preservative due to its broad antimicrobial activity and the history of safe use of k-casein. Kappacin also possesses higher antimicrobial activity in foods with high calcium contents [83]. PIF has a minimum of ~50 mg/mL (CAC 2007) of calcium per serving which would serve to increase the potency of this antimicrobial peptide.

A considerable number of peptides isolated from milk have been reported with various activity and possibilities to prevent the growth of contaminants in PIF [83]. Some may be better candidates for PIF supplementation than others but for all bioactive peptides there are number of hurdles that must be overcome. The natural concentration of peptides in milk is low. Financially viable processes that lead to stable peptides which give reproducible results are needed. To date the commercial production of bioactives has been restricted due to a lack of large scale technologies. Following hydrolysis, milk is often filtered to concentrate the peptides and sometimes fractionated to produce pure peptides [123]. As highlighted by the case of casocidin-1, lengthy and difficult production methods can affect the success of a product. Production difficulties may be offset by potent antimicrobials which can be added in minute amounts to batches of PIF or by generating powders containing antimicrobials that have not undergone the same level of processing (filtering, fractionation) and maintain the ability to inhibit pathogen growth [120]. The case of isracidin where interest waned due to variations in results indicates the importance of batch stability for a successful product. Isracidin also demonstrates the differences between *in vitro* and *in vivo* assays when working with bioactive peptides as they can stimulate the immune system indirectly increasing efficacy in ways not yet understood [97,119]. As peptides designed to prevent the growth of pathogens such as *Cronobacter* would have served their intended purpose before ingestion, stability against stomach enzymes would not be an issue. Metal cations in certain foods can adversely affect cationic peptides though so reactivity with other components present in formula would be have to addressed [82]. Of course, any additive to PIF must be completely safe and not interfere with the organoleptic qualities of the food product.

An AMP generated from a cheap safe source like milk which meets the criteria would prevent the post-rehydration growth of contaminants when added to PIF. Keeping these bacteria at a low level could prevent illness and subsequent deaths and morbidities. An AMP, alone or in synergy with another compound may be the best method to increase the safety of PIF.

### 4.2. Organic Acids

As a group, organic acids primarily include the saturated straight-chain monocarboxylic acids as well as their respective derivatives (phenolic, hydroxylic, unsaturated and multicarboxylic versions) [124]. They have a long history of use in food products as additives and preservatives for prevention of microbial and fungal contamination in food production, processing and storage [125]. In products for human consumption, acetic, sorbic, benzoic and propionic acid constitute the most commonly used acid preservatives due to good solubility, taste and their low toxicity [124]. Although the mechanisms of antimicrobial activities of organic acids have yet to be fully elucidated they are capable of exerting a bacteriostatic or bactericidal effect. These effects are influenced by the physiochemical state of both the organism in question and the surrounding environment. pH is considered the primary determinant given the weak acidic nature of most of these compounds [125,126]. It is assumed that undissociated forms of organic acids can penetrate the cell membrane and once internalised into the neutral pH of the cytoplasm, disassociate into anions and protons effecting the function of macromolecules within the cell [125,126,127]. Exporting excess protons requires ATP and may result in a depletion of cellular energy [127].

Direct acidification of infant formula with lactic acid was reported to be an effective method to prevent the rapid growth of a number of pathogenic bacteria. Infant formula was first fermented with a lactic acid bacteria which negatively influenced the growth of certain pathogens inoculated into the formula. To determine if this effect was due to pH or the presence of the fermenting bacteria, the authors compared the fermented formula with unfermented lactic acid acidified formula. They concluded that the formula acidified with lactic acid had similar bacteriostatic properties as the fermented formula [128]. Due to the increasing interest in anti-*Cronobacter* strategies for infant formula there has been a recent increase in the number of studies investigating the potential of organic acids as protectants.

Recently, nine organic acids were investigated for anti-*Cronobacter* activity. Five of them, namely malic, formic, propionic and citric acid inhibited growth of one or more of the strains investigated on laboratory media. Agar disc diffusion assays showed that propionic acid was the most effective against the 71 strains of *Cronobacter* investigated, producing zones of inhibition (1.5 cm diameter) for all strains except one. From these assays, the authors concluded that the order of inhibition of the organic acids they used against the *Cronobacter* strains was propionic acid > acetic acid > malic acid ≥ citric acid > formic acid. Further assays using the most effective acids, propionic and acetic in a number of food systems, including a nutrition shake designed for infants (PediaSure^®^, Arla Foods amba headoffice Sønderhøj 14, 8260 Viby J., Denmark) showed a bacteriostatic or bactericidal effect at 10 mM and 100 mM, respectively. In this study, hydrochloric acid (pH 4) did not inhibit *Cronobacter* in antimicrobial assays while organic acids at the same pH did indicating that inhibition of growth was not primarily due to pH [126].

In a study published in 2013, Choi *et al.* demonstrated that combinations of caprylic acid, citric acid and vanillin had a destructive effect on a number of *Cronobacter* and *S. Typhimurium*. In their study, the authors spiked reconstituted PIF with the bacteria before adding the antimicrobial compounds. Results indicated that there was a significant synergistic action by the antimicrobials against the pathogens. The authors validated their initial findings by using desiccated cells in order to replicate the type of contamination usually seen in real-world applications. Once again, viability of the *Cronobacter* and *S. Typhimurium* strains was not maintained under the conditions examined. Flow cytometry and electron microscopy indicated plasmolysis and membrane disintegration led to bacterial death [129].

A recent study determined the effect of a number of organic acids on *Cronobacter* growth in both reconstituted PIF and laboratory media as well as the bacteriostatic effect of PIF, slightly acidified with acetic acid (pH 6.0) in combination with a simulated gastric model. The growth characteristics of 30 *Cronobacter* strains at various pH conditions in laboratory medium was first investigated, the results of which indicated that the majority of strains investigated (86%) were resistant to pH 5.0, with zero growth inhibition observed over a 24 h period. The remaining four strains that were sensitive to pH 5.0 were deemed acid sensitive and used in the antimicrobial assays. In laboratory medium acetic, butyric and propionic acids were most inhibitory. The strains investigated were capable of growth at pH 5.5 except when exposed to these acids. The inhibitory effect of these acids in laboratory media was not observed in reconstituted PIF alone but had a synergistic effect when combined with simulated infant gastric conditions as demonstrated by a significant delay in growth of the acid-sensitive *Cronobacter* strains (acetic acid used to acidify the PIF) [130]. The benefits of milk acidification for infants has previously been demonstrated [131]. However, as strong acid acidification is not always well tolerated, interest in the use of organic acids compounds in PIF to control and inhibit the growth of contaminants is rising [125]. Previously, it has been reported that *Cronobacter* strains pre-exposed to highly acidic conditions have a higher tolerance against subsequent acidic exposures [51]. Hence, directly acidifying formula to a lower pH may not be the best option in terms of microbial protection. Zhu *et al.* concluded that milder acidification of formula, in conjunction with infant gastric acid may achieve a protective function similar to direct acidification to a lower pH and will also lead to a product more readily accepted by the infant [130].

With regards to *Cronobacter*, one cause for concern with infant formula acidification is variation in intra-genus susceptibility to the acids, which has been previously demonstrated [126,130]. In this respect, propionic and acetic acids are potent antimicrobials [51,125,126,130,132], possibly due to the greater proportion of acid molecules in an undissociated form relative to other acids, which can increase levels of cytoplasmic acidification in bacteria [127].

From a regulatory perspective, although propionic and acetic acids have GRAS status, they are not currently included in Codex standards for infant formula. Organic acids such as these may have application for the improved safety of PIF alone or in combination with another antimicrobials following further research.

### 4.3. Probiotics and Prebiotics

Probiotics (“live micro-organisms which, when administered in adequate amounts, confer a health benefit on the host” [133]) are increasingly being added to infant formula due to health benefits with which they are associated [134,135]. Prebiotics (“non-digestible carbohydrates which beneficially influence the growth of a specific group of bacteria in gastro-intestinal tract” [136]) such as fructooligosaccharide (FOS) and beta-galacto-oligosaccharide (GOS) inclusion in PIF has also become commonplace due to the purported effect they have on the growth of beneficial bacteria [3]. Various oligosaccharides have been investigated to confirm their prebiotic potential, such as acidic and neutral (GOS) and from pectin hydrolysis, short-and long chain FOS, inulin, and combinations of these substances [15]. The term “Synbiotic” is used when both probiotics and prebiotics are administered together. Early colonization of the gut by commensal microorganisms is associated with a number of beneficial outcomes during infancy, including nutrient provision to the host via hydrolysis of non-digestible food components, modulation of mucosal immunity and the bacteria-mediated production of metabolites such as short chain fatty acids. Of relevance to this article is the fact that the presence of commensal bacteria in the gastrointestinal tract (GIT) is associated with a decrease in pathogenic adherence and colonization of the gut [137].

To date, the outcomes associated with probiotic and prebiotic PIF supplementation are associated with improving stool frequency/consistency, improving gastro-intestinal comfort and reducing the incidence of allergy [134,138]. While an increase in commensal bacteria in the GIT may indirectly lower the risk of infection due their effect on pathogenic adhesion, few studies investigating a direct inhibitory effect on *Cronobacter* have been reported. In one such study strains of *Lactobacillus acidophilus* and *Lactobacillus casei* initially isolated from infant stool samples were screened for antimicrobial activity against *C. sakazakii* strains isolated from infant formula. The authors demonstrated that bacteriocin production by the LAB strains (demonstrated by the use of a cell-free supernatant of the cells) had a significant inhibitory effect on the *C. sakazakii* isolates in reconstituted PIF. Activity was not observed following heat treatment or treatment with gastric enzymes indicating the bacteriocins were heat labile which could make their inclusion in powdered milk products difficult. The inclusion of live *L. acidophilus* and *L. casei* strains in reconstituted PIF also had an antimicrobial effect on certain strains of *C. sakazakii* while the CFU/mL of the LAB strains increased by about 2 logs (over a 6 h period). The authors concluded that further research was required to decide the best way to incorporate either the antimicrobial metabolites generated by the LAB strains, or the strains themselves into a PIF product [139].

A number of studies have indicated that prebiotic compounds can influence bacterial adhesion to the GIT [140,141]. The ability of an oligosaccharide to block adhesion depends on a structural similarity between carbohydrate binding sites usually recognised by pathogenic bacteria and the prebiotic. The prebiotic then binds to adhesins on the pathogen thereby inhibiting adherence to host epithelial cells [142]. With specific regard to *Cronobacter* adhesion inhibition, Quintero *et al.* [110] found that either singly or in combination GOS and polydextrose (PDX) significantly reduced the levels of adherence *in-vitro* on two cell-lines representing the gut epithelium. Interestingly, both GOS and PDX are two oligosaccharides commercially used in infant formulae. It should be noted that the effects *in-vivo* were not investigated and the study used levels of GOS and PDX at higher concentrations than that found in infant formula.

Although initial results are promising, further studies are required to confirm if probiotics and/or prebiotics could effectively reduce *Cronobacter* infections related to PIF through adhesion inhibition or a direct antimicrobial action. Due to the current interest in *Cronobacter* further studies may lead to the discovery of GRAS strains with potent anti-*Cronobacter* activity. These strains would have to be able to survive the harsh processing and shelf-life conditions associated with PIF. If this inhibitory action was due to bacteriocin production, efficacy would have to be confirmed *in-vivo* as expression of bacteriocins by bacteria can differ from *in-vitro* conditions [139]. Economic factors would also be an issue particularly with regard to prebiotic oligosaccharides. For example, even if a human milk oligosaccharide was found to inhibit *Cronobacter* adhesion, its commercial use would be difficult to implement.

### 4.4. Alternate Methods of PIF Sterilization under Research

Current knowledge dictates that total sterilization of PIF is only possible using irradiation, but as previously mentioned, this is forbidden by the Codex Alimentarius guidelines on PIF production. In PIF, *Cronobacter* is present in a dessicated state and the high doses required to inactivate it would have severe adverse effects on the organoleptic quality of the product [13,18,58]. Researchers are currently investigating methods to improve the microbiological safety of PIF. A study performed in 2007 reported the effect gamma irradiation against *Cronobacter* strains. The authors found that a dose of 1 kilogray (kGy) effectively reduced PIF bacterial concentration in lab media and reconstituted PIF. The irradiation dose required to inactivate the bacteria in unreconstituted PIF was orders of magnitude higher (9 kGy). The authors attributed this to the presence of water in the reconstituted form indirectly damaging the pathogens [143]. A similar trial by Lee *et al.* (2007) indicated that *Cronobacter* survives gamma irradiation better in unreconstituted PIF. This trial also monitored possible regrowth of the strain in reconstituted PIF. The data generated indicated that a temperature of at least 10 °C was required to allow for regrowth of the microorganism [144]. Along with gamma ray, electron–beam is the most common type of ionizing radiation used in food processing. In a study, published in 2008, researchers investigated electron-beam radiation as a method to sterilize a powdered weaning food. The results indicated that *Cronobacter*, *B. cereus* and *S. typhimurium* strains could be eliminated from the food by irradiation at 16, 8 and 8 kGy respectively, indicating that *Cronobacter* was the most resistant organism tested. Indeed, the study described how the irradiation dose required to eliminate the pathogen unacceptably affected the sensory qualities of the weaning food [145].

Another method of unreconstituted PIF sterilization was described in 2010. In this study the use of supercritical carbon dioxide (SC-CO_2_) in conjunction with a heat-treatment (63–73 °C) reduced *Cronobacter* numbers in a dry product. SC-CO_2_ is a non-flammable, non-toxic and chemically inert process. The authors concluded that the method they outlined effectively reduced levels of the bacteria without adverse effects on the final product. Although the process was relatively complex a simplified and larger capacity method could feasibly be used to improve the microbiological quality of PIF. It should be noted that the results indicated that an increase in temperature added to the bacteriocidal effect [146].

Ultraviolet (UV) radiation is capable of penetrating bacterial cell membranes and consequently damaging DNA. A recent study indicated that a UV treatment had bacteriocidal effects on a *Cronobacter* strain. The authors findings indicated that UV radiation treatment for 25 min reduced the population of the microorganism in dry PIF by 1.38 log 10 CFU/gram. Complete inactivation was not possible due the effect the food matrix had on the process [147]. Indeed, although UV radiation is well established for use in water and air sanitation, and liquid food pasteurization, it’s effectiveness for powder food sterilization is still under investigation [147,148]. A further reduction in viable *Cronobacter* cells was observed when the UV radiation process was combined with a thermal treatment (reconstitution with water at 55, 60 or 65 °C). The authors postulated that a decrease in cellular heat resistance may have occurred due to UV radiation mediated cell injury [147]. Ha *et al.* recently described the anti-*Cronobacter* effect of near-infrared radiant (NIR) heating, in combination with UV irradiation in PIF. NIR is a form of electromagnetic energy that can cause heating if absorbed [148]. The benefit this process has over classical thermal-treatments is that it is unaffected by the air which surrounds the treated product [149]. The study indicated that a synergy between the two processes led to a bacteriocidal effect greater than the sum of separate NIR and UV irradiation treatments. A propidium iodide uptake assay indicated that disruption of the bacterial cell membrane was most responsible for the antimicrobial effect observed. The study indicated that combined NIR-UV treatment led to a 2.79 log/unit CFU reduction in *C. sakazakii* numbers. The authors concluded that up-scaling of this process to industrially relevant levels would be practical [148].

Currently the use of technologies such as magnetic fields and ultra-high pressure are at early stages of development and their effectiveness has not yet been established [60]. The use of microwave technology on reconstituted PIF has proven effective at reducing numbers of *Cronobacter* cells in what is believed to both a non-thermal electromagnetic radiation effect and a direct thermal effect on the bacteria. In one trial, microwaving reconstituted PIF for 90 s at 93 °C effectively reduced an inoculum of 100 CFU/mL *Cronobacter* cells to zero [150]. However, the effect of this treatment on the nutrient value of the product would likely be an issue. There would also likely be adherence issues on the part of caregivers with this treatment.

## 5. Conclusions

The microbiological integrity of PIF has been re-examined over the past decade in part due to the emergence of *Cronobacter*. A number of guidelines and regulatory changes have been published [6,8] in order to aid caregivers and manufacturers of PIF and PIF ingredients reduce the chance of catastrophic contamination by pathogenic bacteria. Strict adherence to these guidelines will reduce incidences of PIF mediated infection [53]. However, it is likely that on certain occasions these recommendations will not be followed either through negligence or incompetence. When this occurs the inclusion of antimicrobial solutions may help prevent illness. Natural antimicrobials, in particular those with GRAS status are of interest due to consumer concerns regarding chemical additives.

With specific regard to *Cronobacter*, the recent increase in research on this genus has led to greatly improved detection methods and knowledge of important characteristics of individual species. A significant challenge is the identification of specific virulence factors related to particular species or strains [60]. Molecular methods are increasingly used to reliably and rapidly trace sources of infection and to study diversity between bacterial genomes [35]. Increasingly, Multilocus sequence typing (MLST) is used to understand the diversity and evolution of pathogenic bacteria. A MLST scheme for *Cronobacter* [151] is available online (http://pubmlst.org/cronobacter/) [152] and currently contains ~900 isolates. Researchers group sequence types elucidated by MLST into clonal complexes decided by similarities to a central allelic profile. The *C. sakazakii* clonal complex ST4 has been frequently isolated from PIF processing plants, milk powder processing facilities and PIF itself [35,45,57,153] and is associated with cases of meningitis. Control of the ST4 lineage could reduce infant exposure to particularly virulent strains [35]. Information derived from the study of strains from this clonal complex may also allow researchers to develop specific methods to prevent growth of virulent strains in various environments.

A multi-directional approach may be the most effective. For PIF producers, continued research into effective decontamination methods coupled with improved training and education of staff and strict implementation of good manufacturing practices are required. Efforts to educate medical staff and infant caregivers regarding proper handling and storage of the product should be increased [60]. Consumers should also receive clear and accurate information regarding the risk of infection associated with various formulae so that they may make informed decisions regarding how to feed the infant [9]. Finally, the inclusion of antimicrobials either singly, or in synergy with other compounds or processes will help lower incidences of infection.
